# Retraction: Spermidine/spermine N^1^-acetyltransferase regulates cell growth and metastasis *via* AKT/β-catenin signaling pathways in hepatocellular and colorectal carcinoma cells

**DOI:** 10.18632/oncotarget.28799

**Published:** 2025-12-31

**Authors:** Cong Wang, Ping Ruan, Ying Zhao, Xiaomin Li, Jun Wang, Xiaoxiao Wu, Tong Liu, Shasha Wang, Jiuzhou Hou, Wei Li, Qian Li, Jinghua Li, Fujun Dai, Dong Fang, Chaojie Wang, Songqiang Xie

**Affiliations:** ^1^Institute of Chemical Biology, College of Pharmacy, Henan University, Kaifeng, 475004, China; ^2^The Key Laboratory of Natural Medicine and Immuno-Engineering, Henan University, Kaifeng, 475004, China; ^*^These authors have contributed equally to this work

**This article has been retracted:** Oncotarget has completed its investigation of this paper, and has found and confirmed multiple instances of image duplication and overlap. These issues are detailed below:

- Figure 4C Transwell Invasion Assay: The images for SSAT#2 (a), Hep G2 (b), and HCT116 (c) overlap with the images of SSAT#1 (a), Vector (b), and Vector (c), respectively.
- Figure 5A(a) Wound Healing Assay: Overlaps exist between the non-treated and siControl-transfected Bel7402 cells at the 0 h and 24 h timepoints.
- Figure 5C(a) Transwell Invasion Assay: Images of Bel7402 and siSSAT#1 in Bel7402 group partially overlap with the SSAT#2 and polyamine plus SSAT#1 images in Hep2 group in Figure 9C, respectively.
- Figure 7A(b) Western Blot: The image for b-actin is duplicated with the b-actin WB in Figure 8A(c).
- Figure 7B(a): The b-catenin image duplicates SSAT in Figure 1B(b).
- Figure 7C(a): The b-actin image duplicates the Lamin B1 image in Figure 7C(b).
- Figure 8C Transwell Invasion Assay: The image of SSAT#2 in the SMMC-7721 group partially overlaps with the image of SSAT#2 in the Hep G2 group.

While the authors provided corrected versions of Figures 4, 5, and 8, the original, unmodified images from the original experiments were not supplied to support these corrections.

Due to the substantial number of duplications and overlaps confirmed in the manuscript, and the absence of original supporting data, the Editorial decision has been made to retract this paper.

Original article: Oncotarget. 2017; 8:1092–1109. 1092-1109. https://doi.org/10.18632/oncotarget.13582

